# Supplementary material for: Targeted integration of extrachromosomal arrays in C. elegans using PhiC31 integrase
Source: Sci Adv. 2026 Oct 7;12(41):eaef6902. doi: 10.1126/sciadv.aef6902 (PMC13644748; doi:10.1126/sciadv.aef6902)
Supplement: Supplementary file 1 [file sciadv.aef6902_sm.pdf]

Science Advances
AAAS

Supplementary Materials for

Targeted integration of extrachromosomal arrays in *C. elegans* using PhiC31 integrase

Matthew S. Rich *et al.*

Corresponding author: Erik M. Jorgensen, jorgensen@biology.utah.edu

Sci. Adv. **12**, eaef6902 (2026)
DOI: 10.1126/sciadv.aef6902

The PDF file includes:

Figs. S1 to S5
File S3
Legends for files S1, S2, S4 and S5
Legends for tables S1 to S7

Other Supplementary Material for this manuscript includes the following:

Files S1, S2, S4 and S5
Tables S1 to S7

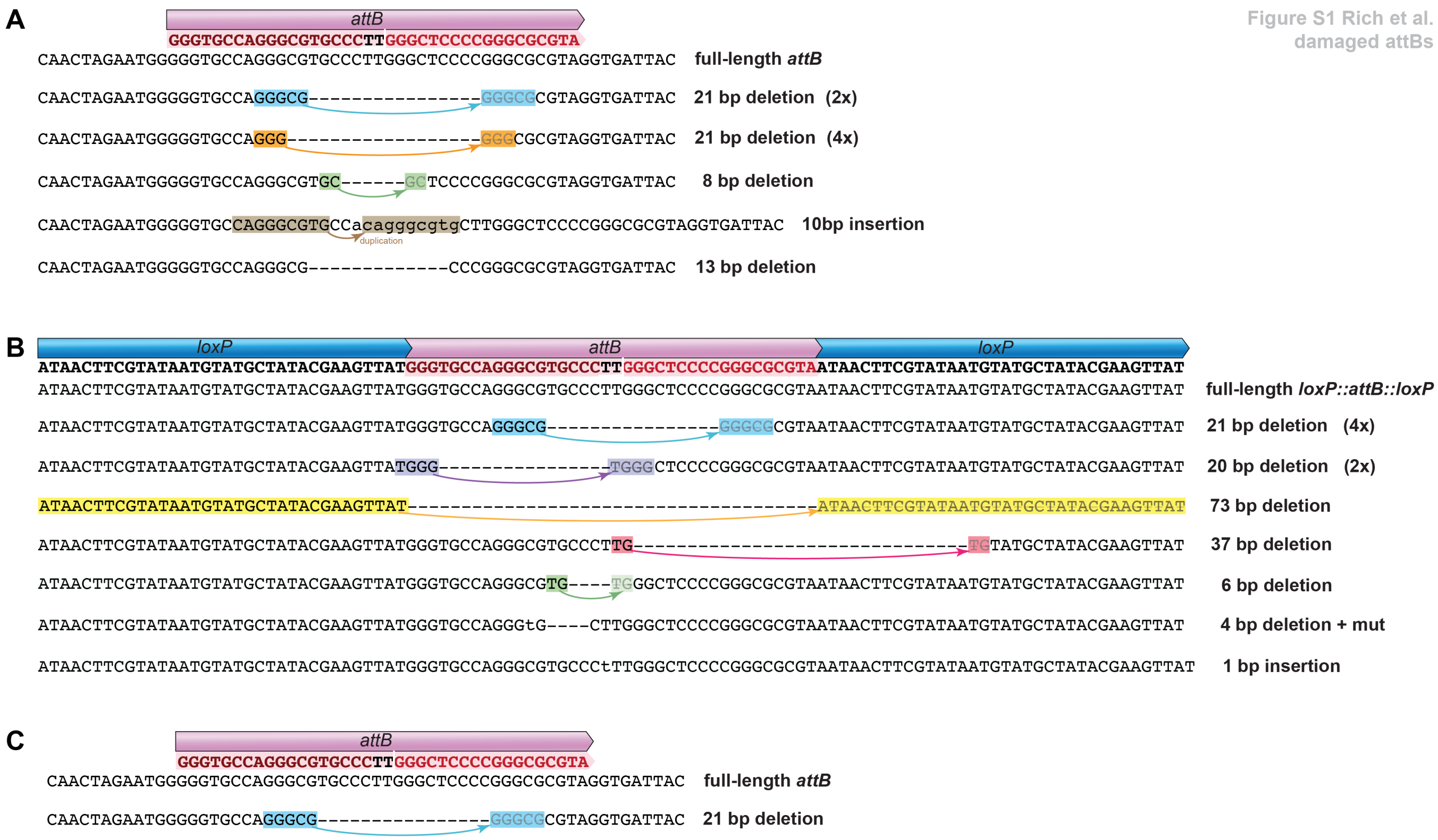

Figure S1. Mutated *attB* sites. We sequenced the *attB* sites in strains that did not yield integrants. Each line is a different mutation to the *attB* site, with a description and whether it was isolated multiple times shown to the right. Most mutations were consistent with a microhomology-mediated repair causing a deletion. These repairs are annotated with colored highlights showing the relevant microhomology in which gray residues are now absent. (A) Mutated *attB* sites from attempted integrations into *oxSi1337*, (B) into *oxSi1411*. (C) The mutation found in *oxSi1337* after long-term propagation with PhiC31. This mutation was also found in (A) and (B).

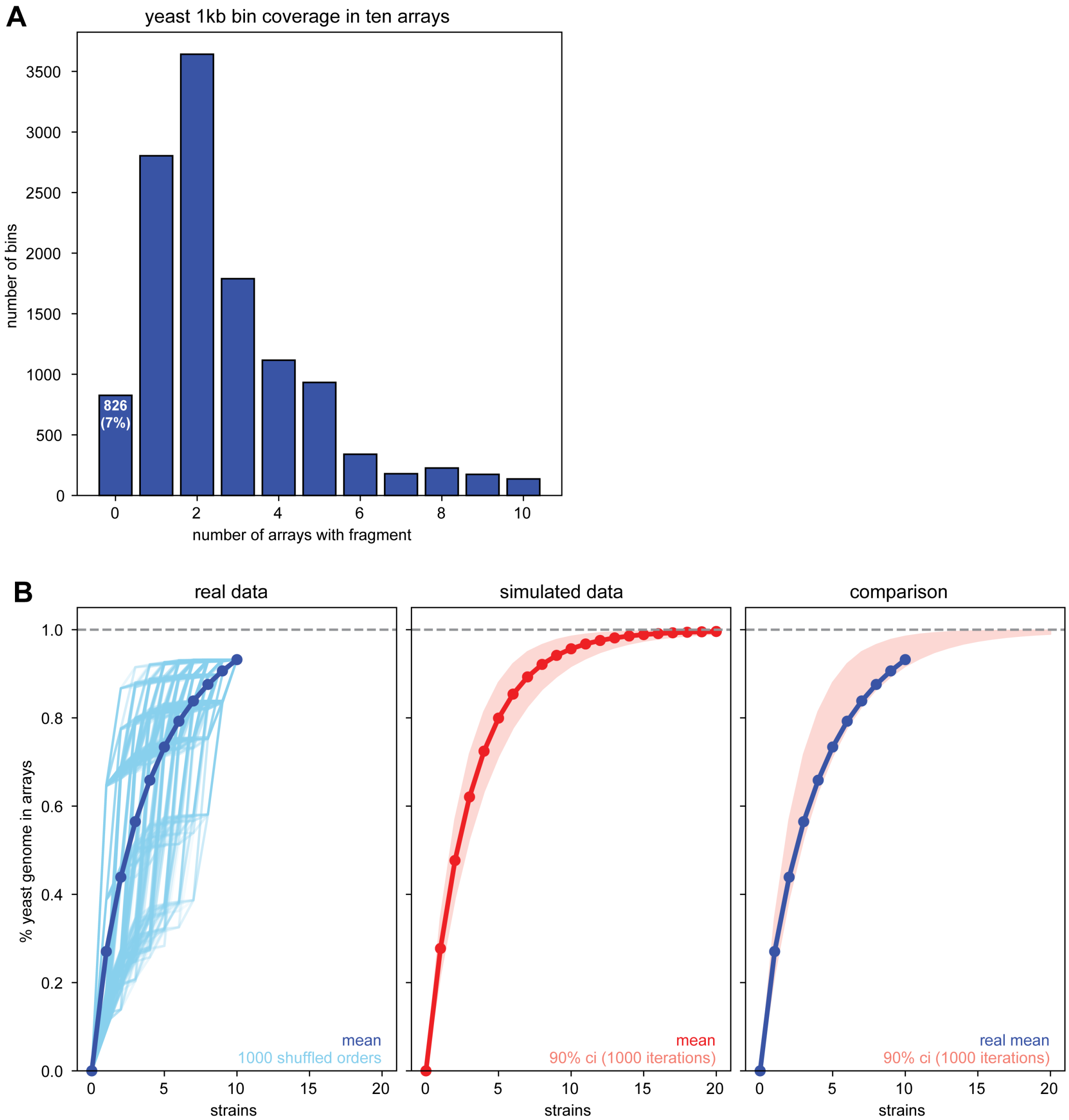

Figure S2. Yeast fragments in integrated arrays. (A) Number of times yeast genomic fragments are found in 10 sequenced arrays. The yeast genome was split into 1000 bp bins, and whether the sequence in each bin was present in an array was determined by short-read whole genome sequencing. 93% of the yeast genome is found in at least one of the ten arrays. (B) Saturation of yeast genome in 10 arrays. (Left) Average saturation (dark blue) was calculated using a Monte Carlo simulation shuffling the order that arrays were added to the cumulative plot (light blue lines). For example, the left-most light blue line is the order in which arrays containing the most yeast DNA were the sampled first, and the right-most line is the order in which the arrays containing the least yeast DNA were sampled first. (Middle) An expected distribution for incorporation of yeast fragments into arrays was created using a Monte Carlo simulation (see Methods). (Right) Overlay of the real mean saturation with the 90% confidence interval from simulated data. The real arrays (blue line) grow slower than expected (but within the confidence interval). This is likely due to the yeast rDNA being overrepresented in the injected DNA (~150 copies) compared to the reference genome (2 copies).

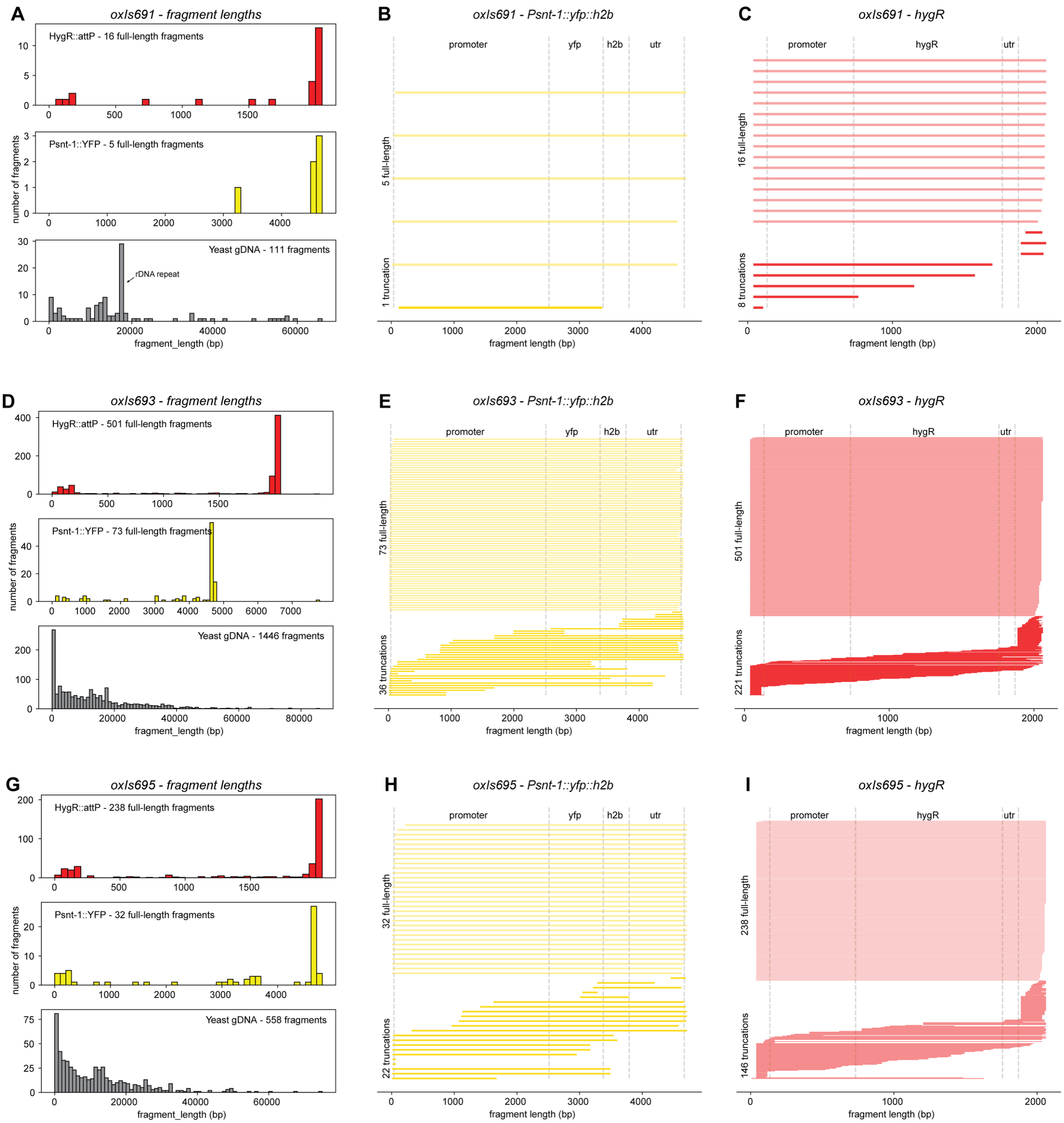

Figure S3. Transgene fragments in assembled integrated arrays. Contigs from *oxIs691*, *oxIs693*, and *oxIs695* were aligned to the yeast genome, P*snt-1::YFP*, and *HygR::attP* references. Mapped fragments were filtered for those having a mapping quality > 0. For *HygR::attP* and P*snt-1::YFP*, being called as a “full-length” fragment required a mapping length of at least 95% of the reference length.

For *Psnt-1::YFP* and *HygR* fragment plots, each mapped fragment is a horizontal line, with full-length fragments on top and truncated fragments on the bottom of the plot. Boundaries between functional regions are shown by vertical dashed lines. *oxIs691:* (A) fragment length distributions; (B) *Psnt-1::YFP* fragments; (C) *HygR* fragments. *oxIs693:* (D) fragment length distributions; (E) *Psnt-1::YFP* fragments; (F) *HygR* fragments. *oxIs695:* (G) fragment length distributions; (H) *Psnt-1::YFP* fragments; (I) *HygR* fragments.

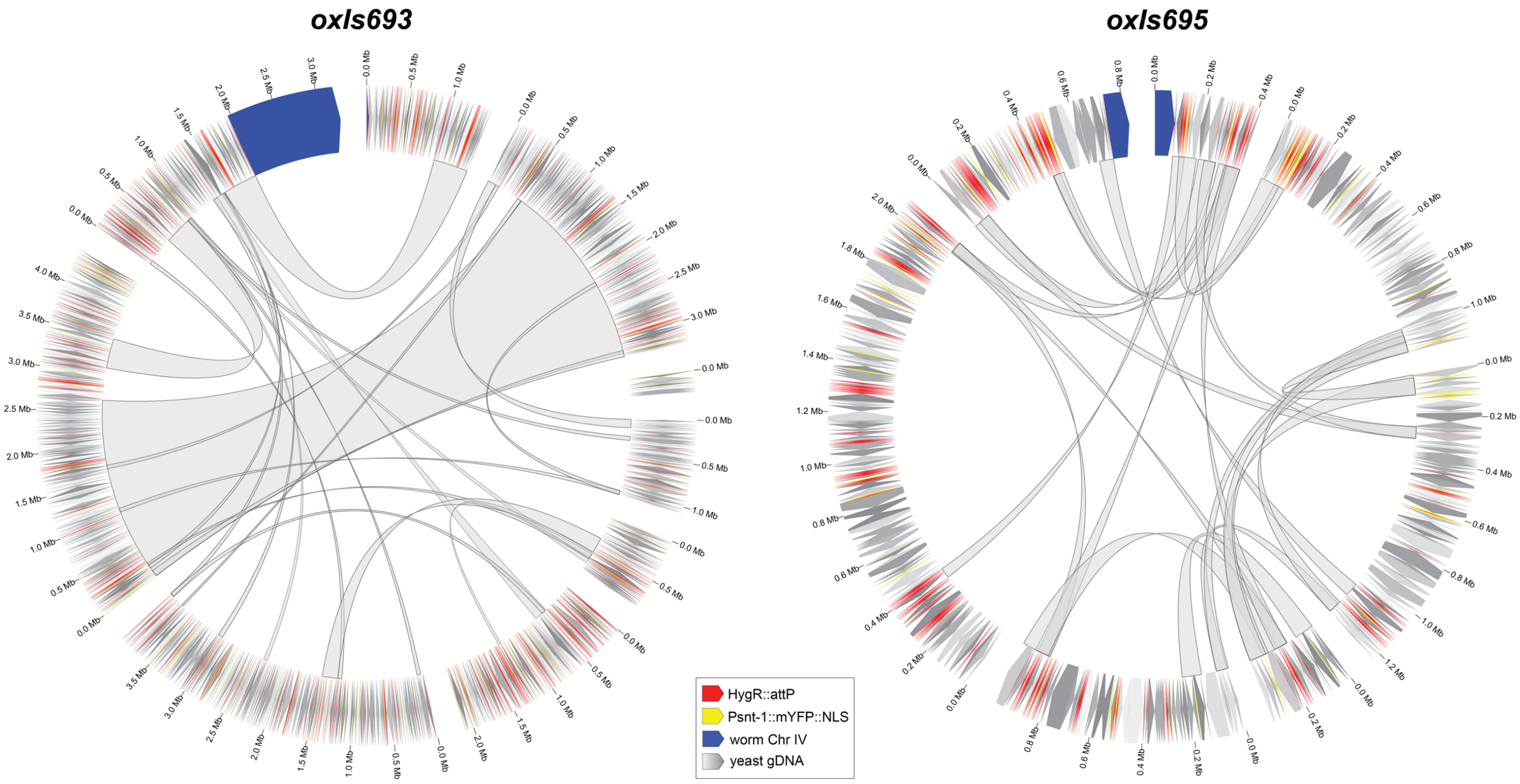

Figure S4. Large repeats found in integrated arrays. Repeats longer than 50 kb that are found on multiple contigs are plotted as gray arcs in Circos plots for *oxIs693* (left) and *oxIs695* (right). Around the outside of the plots, the annotated array contigs are plotted as in Figure 4. Gaps between contigs are caused by repeats that were too long for a sequencing read to span. Reads used for assembly were all at least 50 kb but no longer than 250 kb. Both arrays have 20 of these repeats.

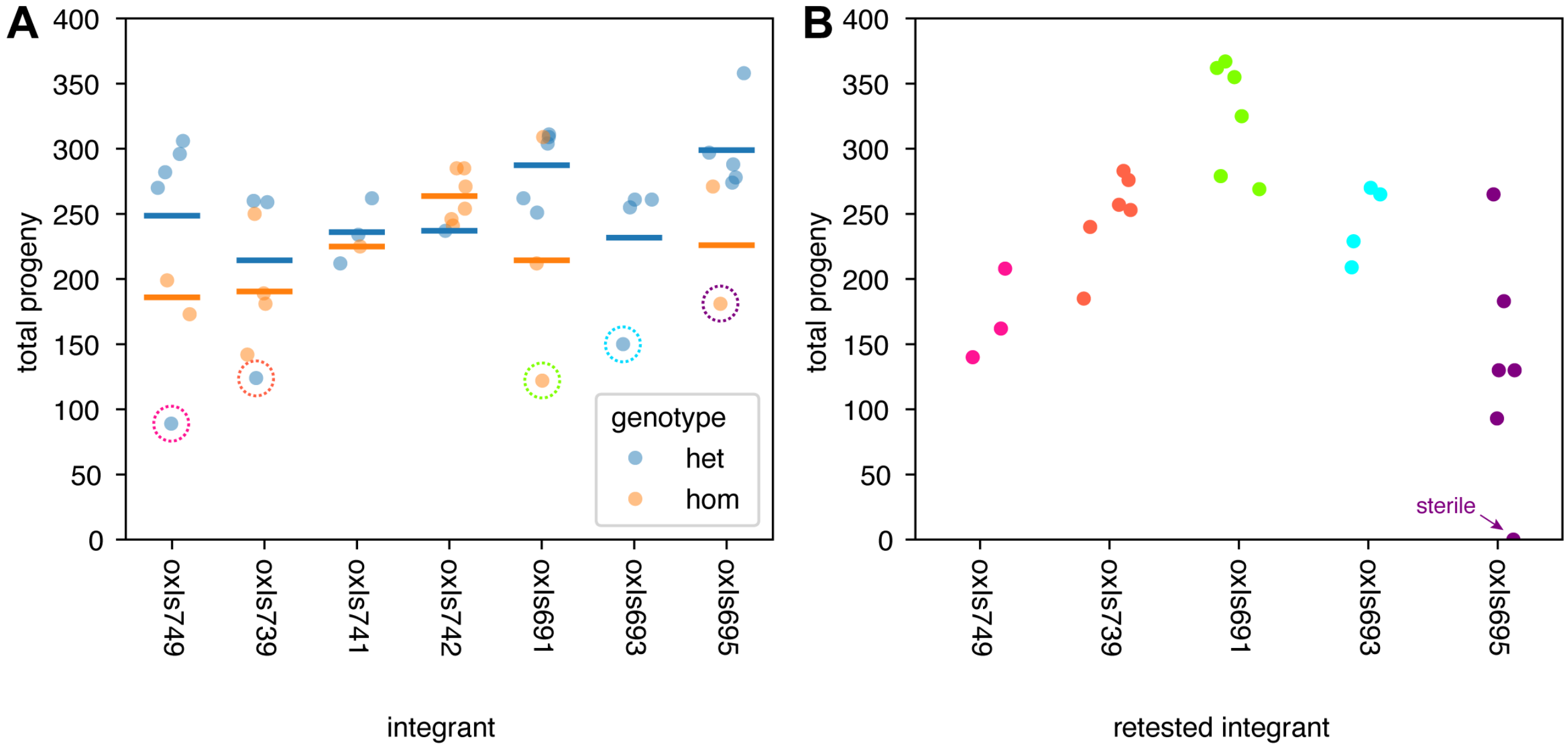

Figure S5. Fecundity of integrated array strains. (A) Array strains were outcrossed and their progeny counted. Orange: animals heterozygous for the array; Blue: animals homozygous for the array. Each point is the total progeny counted for a single animal, bars are means of the genotype. Circles indicate outliers with low fecundity. Progeny from these animals were retested in (B) (colored circles in A; correspond to colors in B). (B) Retesting fecundity in outliers. Progeny from circled animals in (A) were retested to determine whether their low-fecundity was hereditable. One retested line from *oxIs695* was sterile.

Targeted integration of extrachromosomal arrays in *C. elegans* using PhiC31 integrase

Matthew S. Rich, Ryan Pellow, Yichen Zhang, Adam Hefel, Ofer Rog, Erik M. Jorgensen*

File S3

Annotated protocol for PhiAT

A step-by-step protocol for the method including notes

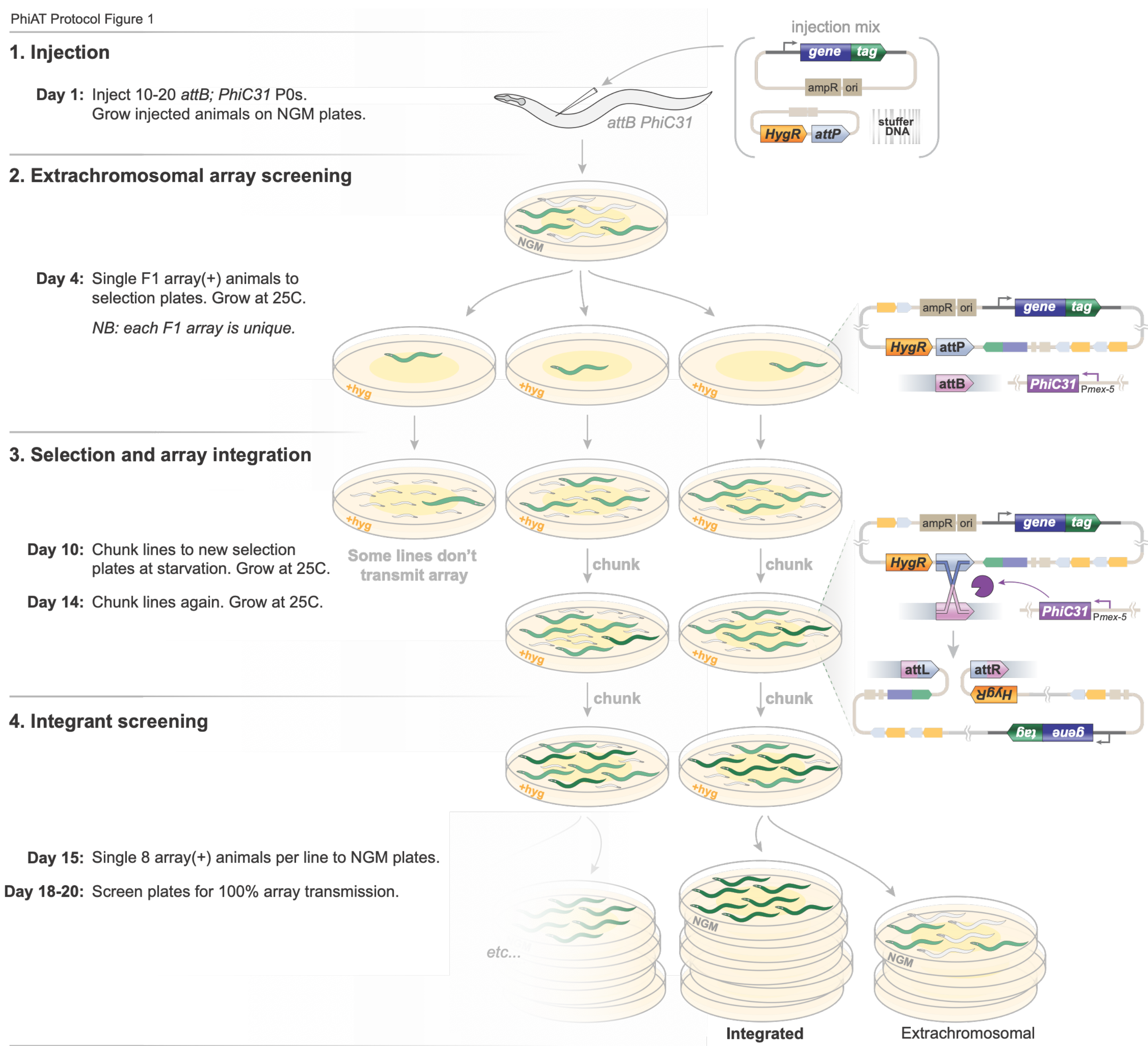

PhIAT protocol [1]

0. Creating injection strain [2]

Day -10 Cross *attB; RFP Him* males to *balancer; PhiC31::mNG* hermaphrodites [3].

Day -7 Single *attB/balancer; PhiC31::mNG/+; RFP Him/+* hermaphrodite F1 cross-progeny.

Day -4 Single 8x *balancer(-) RFP(-) PhiC31::mNG(+)* hermaphrodites to HB101 plates. Grow at 15C.

Day -1 Screen adults for homozygous *PhiC31::mNG(+)* lines. Keep one line at 15C [4].

1. Injection

Day 0 Grow *attB PhiC31* strain on HB101 plates at 15C [4].

Day 1 Make injection mix that includes transgenes, selection markers, and *attP*. See note (5) below for details.

Inject 10-20 animals [6].

Place injected animals on HB101 plates. Incubate at 25C.

2. Extrachromosomal array screening

Day 4 Single F1 array(+) animals to new HB101 plates, supplemented with drug selection if necessary [7]. Incubate plates at 25C.

Note: all F1 arrays are unique. Single many to get diverse integrants.

3. Selection

Day 10 Chunk surviving lines to new selection plates [8].

Day 14 Chunk surviving lines to new selection plates.

4. Screening [9]

Day 15 Single 8 array(+) animals per line to **NGM** plates [10].

Day 18-20 Screen plates for 100% array transmission. These are integrants [11].

5. Outcross

Day 21 Cross integrant to N2 [12] males to outcross and remove PhiC31.

PhIAT protocol notes:

1 See PhIAT protocol illustrated steps (Protocol Fig. 1)

2 PhiC31 will cut an *attB* site without an *attP* present, eventually mutating the *attB* and eliminating integration. As such, for long-term storage, we maintain *attB* and *PhiC31* strains separately. Before integration injections, the strains are crossed to reconstitute an injectable integration strain.

3 The strains you cross depends on which *attB* you want to integrate at. To simplify reconstitution crosses, we made PhiC31 strains containing *attB* balancers and *attB* strains containing fluorescently-marked Him alleles:

attB strain	x	balancer + PhiC31 strain
EG10541 *attB I; RFP him-8 IV*	x	EG10478 *Bal[unc-40(-), YFP] I; PhiC31[mNG] II*
EG10542 *attB II; RFP him-8 IV*	x	EG10570 *Bal[dpy-10(-) unc-52(-), GFP] II / mnDf61[lethal]; PhiC31[mNG] IV*
EG10543 *attB IV; him-5 RFP V*	x	EG10473 *PhiC31[mNG] II; Bal[unc-5(-), YFP] IV*

For example, to make an injection strain for the *attB* site on chromosome IV, you would follow the cross shown in Protocol Fig. 2.

Heterozygotes can also be injected and will yield integrants. In this method, we recommend pooling injected animals and screening the whole pool for integrants.

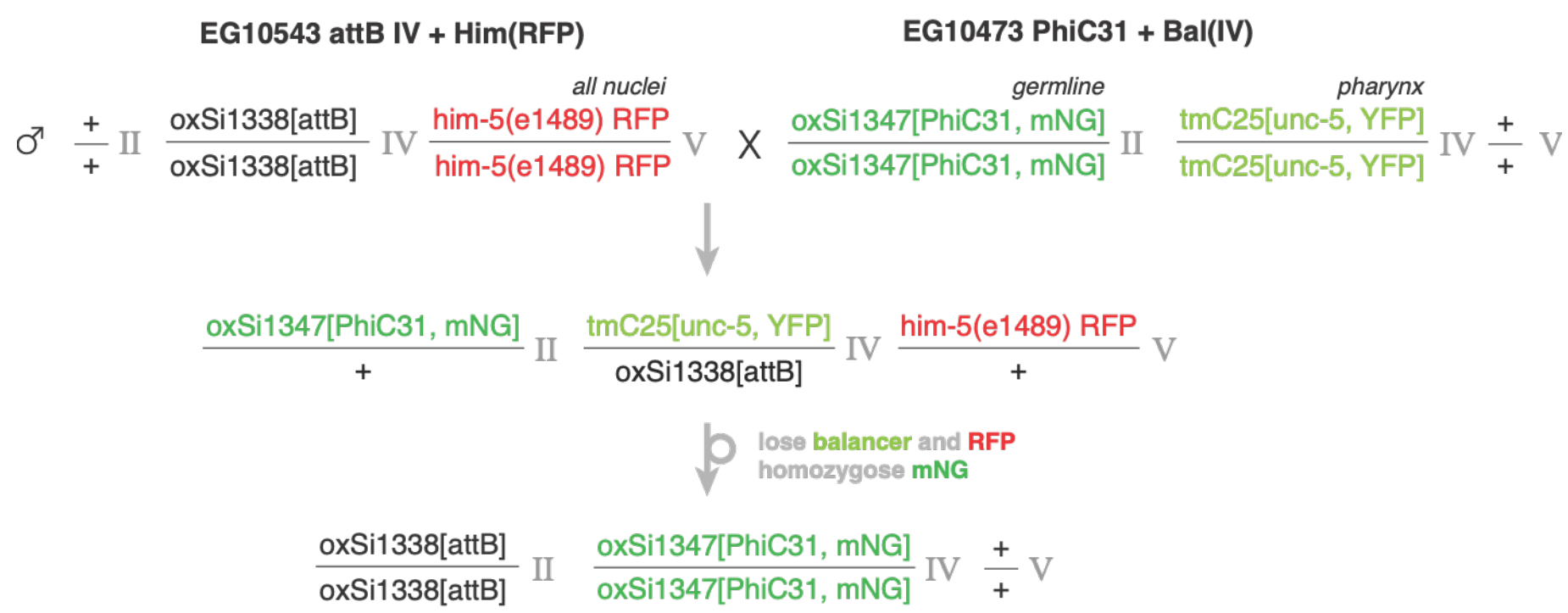

Protocol Figure 2. Example injection strain reconstitution cross.

4 *attB* mutagenesis occurs at a non-trivial (but currently unknown) rate in the germline of an *attB PhiC31* strain. As such, we recommend propagating injection strains only when necessary to limit the number of generations they go through (and therefore limit the frequency of mutated *attB* sites in the population).

5 A PhIAT injection has 4 components: your transgenes, a selectable marker, *attP* sites, and a fluorescent array marker. Arrays can be made either with linear DNAs (in which *attP* sites are appended by PCR to selection plasmids) or plasmid DNAs (in which pMR903 is coinjected along with transgene and selection plasmids). Note: we usually find that expression from linear arrays is much stronger than in plasmid arrays, and so the relative amount of transgene plasmids should be reduced, sometimes over ten-fold. See Tables 2 and 3 for examples; these injections yielded similarly-bright arrays.

DNA	ng/ul
HygR::attP PCR (from pCFJ782)	2.5
Pmlc-1::TagRFP PCR (from pSEM233)	1
DNA ladder	100

Table 2. Linear PhIAT injection

DNA	ng/ul
pMR903 (attP)	6
pSEM233 (Pmlc-1::TagRFP)	11
pCFJ782 (HygR)	10
DNA ladder	75

Table 3. Plasmid PhIAT injection

6 The number of injected animals may vary depending on injector skill. We aim for having ~50 F1 arrays, and routinely get this many arrays by injecting about 10 animals in a single gonad arm.

7 We have integrated arrays using hygromycin B, neomycin/G418, and puromycin drug selections, as well as *unc-119(+)* rescue (see Table 4 for drug concentrations and resistance plasmids).

For drug selections, top-plate 500ul of drug stock on a 4cm plate. Let the plate dry with the lid off (this usually takes about 30-60 minutes) before storage. We purchase our drugs from Gold Bio.

Selection	Stock conc.	Plasmid
Hygromycin B	4 mg/ml	pCFJ782
Neomycin/G418	25 mg/ml	pCFJ594
Puromycin	10 mg/ml + 0.1% Triton100	pCFJ744
unc-119(-)	n/a	pMR201

Table 4. Selections and their plasmids.

8 In our hands, 30-50% of F1 arrays are transmitted to the next generation.

9 The primary method we use to isolate integrants is screening for homozygous segregation of a fluorescent marker. Integrants can also be identified by PCR, as the *attB+attP -> attL+attR* recombination is predictable (unpublished). ApE has a recombination tool that allows for testing these recombinations *in silico* and designing primers to genotype them (https://jorgensen.biology.utah.edu/wayned/ape/).

10 The number of animals to single for screening is arbitrary. We recommend eight for two reasons: first, it's a large enough set to not miss relatively low-frequency integrants, and second, a stack of eight plates fits well in our incubators. Integrants will be isolated from using fewer plates per line, but some might be missed. For screening large numbers of lines, we have used GelDrops (PMID: 40985011) on the lids of 96-well plates.

11 Because PhIAT uses a selection, any array that transmits near 100% will increase in population frequency during selection. The primary form of false-positives during screening are extrachromosomal arrays that transmit near 100%. To get rid of these, we grow putative integrant plates for 2-3 generations after singling; after two generations a plate with a high-transmission array will have many array(-) animals, while a true integrant will have zero.

12 We recommend outcrossing integrants to remove the PhiC31 transgene and limit off-target mutations in the strain background. While this cross can be done with a wild-type strain, we recommend using a visible mapping marker to confirm the array inserted at the correct site. Some good mapping markers are:

- *oxSi1337* I@-5.3 vs *dpy-5(e61)* I@0
- *oxSi1270* II@0.8 vs *dpy-10(e128)* II@0
- *oxSi1338* IV@1.4 vs *unc-22(e66)* IV@5.5

Strains list (see Table S1 for full list of strains.)

PhiC31 strains

strain	description	genotype
EG10408	PhiC31 II	oxSi1347[Pmex-5::PhiC31(PATC)::sl2::mNeongreen::glh-2UTR *jsSi1579] II
NM5406	PhiC31 IV	jsSi1623[loxP, Pmex-5::phiC31::SL2::mNeongreen::glh-2UTR, FRT3] IV
EG10476	PhiC31 with balancer for attB I	tmC20[unc-14(tmIs1219)] I ; oxSi1347[Pmex-5::PhiC31(PATC)::sl2::mNeongreen::glh-2UTR, *jsSi1579] II
EG10570	PhiC31 with balancer for attB II	mnC1[dpy-10(e128) umnIs32(Pmyo-2::GFP) unc-52(e444)] / mnDf61[lethal] II ; jsSi1623[loxP Pmex 5::PhiC31::SL2::mNeonGreen::glh-2UTR FRT3] IV
EG10473	PhiC31 with balancer for attB IV	oxSi1347[Pmex-5::PhiC31(PATC)::sl2::mNeongreen::glh-2UTR, *jsSi1579] II ; tmC25 [unc-5(tmIs1241[Pmyo-2::YFP])] IV

attB strains

strain	description	genotype
EG10469	attB near ttTi4348 I	oxSi1337[attB, *ttTi4348] I
EG10470	attB near ttTi5605 II	oxSi1270[attB, *ttTi5605] II
EG10471	attB near cxTi10816 IV	oxSi1338[attB, *cxTi10816] IV
EG10541	attB I + RFP him-8 IV	oxSi1337[attB] I ; oxTi404[Peft-3::tdTomato::H2B::unc-54 cb-unc-119(+)] him-8(ox1602) IV
EG10542	attB II + RFP him-8 IV	oxSi1270[attB] II ; oxTi404[Peft-3::tdTomato::H2B::unc-54 cb-unc-119(+)] him-8(ox1602) IV
EG10543	attB IV + him-5 RFP V	oxSi1338[attB] IV ; him-5(e1490) oxTi405[Peft-3::tdTomato::H2B::unc-54 cb-unc-119(+)] V
EG10481	CyOFP(loxP-attB-loxP) I	oxSi1411[Pelo-5::CyOFP(loxP-attB-loxP), *oxTi185] I ; unc-119(ed3) III
EG10567	CyOFP(attB) I	oxSi1451[Pelo-5::CyOFP(attB)::rps-1UTR, *jsSi1727] I
EG10568	CyOFP(attB) II	oxSi1452[Pelo-5::CyOFP(attB)::rps-1UTR, *jsSi1726] II
EG10569	CyOFP(attB) IV	oxSi1453[Pelo-5::CyOFP(attB)::rps-1UTR, *jsSi1986] IV

Plasmids and primers lists (see Table S2 for full list of plasmids and primers)

attP and selection plasmids

plasmid	description
pMR903	197bp attP
pMR791	39bp attP (low efficiency)
pCFJ594	G418R
pCFJ707	PuroR
pCFJ782	HygR
pMR201	Cbr-unc-119(+)

fluorescent plasmids used in manuscript

plasmid	description
pSEM233	Pmlc-1::TagRFP-T::tbb-2UTR (pan-muscle, cytosolic)
pMR196	Psnt-1::YFP::histone::unc-54UTR (pan-neuronal, nuclear localized)

primers	sequence (5'-3')	description
M13_F	TGTAAAACGACGGCCAGT	
attP::M13R	CGCCCCCAACTGAGAGAACTCAAAGGTTACCCCAGTTGGGGcaggaaacagctatgaccatg	attP (capitalized) appended to M13R

Supplemental Files and Tables List

File S1: All integration experiment data (.xlsx).

exptinfo_table: metadata for all injections

results_table: integration results dataset per F1 line

other tables: per injection results

File S2: Plasmid sequences (.gb)

File S3: Annotated protocol for PhIAT (pdf). A step-by-step protocol for the method, including notes.

File S4: Analysis Jupyter notebooks (.ipynb)

integration_rates.ipynb: for building Figure 1B

array_size_estimates.ipynb: for estimating array sizes from short read sequencing

coverage_saturation.ipynb: estimating coverage of yeast genomic DNA in arrays

plot_array_contigs.ipynb: plot annotated sequences in arrays

File S5: Array contig sequences (.fa)

Supplementary Tables File (.xlsx)**:**

Table S1: *Strains*. All strains used in this manuscript.

Table S2: *Plasmids*. All strains used in this manuscript.

Table S3: Primers and guide RNAs. All strains used in this manuscript.

Table S4: *Injections*. DNAs and strains used in each injection.

Table S5: *Array size estimates*. Array size was either estimated using short-read sequencing or determined from long-read nanopore assemblies.

Table S6: *Long-read sequencing statistics.* Statistics from Nanoplot and Flye for long-read sequencing.

Table S7: *Array stability results.* Array lines were tested for their stability across generations, through crosses in males and whether they caused a fecundity defect (see Figure S5).
